# Supplementary material for: Are carbon emissions trading and green financial instruments synergistic? -Comprehensive quantitative research based on content analysis
Source: PLoS One. 2024 Mar 7;19(3):e0298601. doi: 10.1371/journal.pone.0298601 (PMC10919601; doi:10.1371/journal.pone.0298601)
Supplement: S1 Data — (ZIP) [file pone.0298601.s001.zip › Data/地级市Z_F工作报告.zip!/╜¡╦╒/╠⌐╓▌╩╨/└┤╫╘╣1⁄2╓┌║┼í╛┬φ┐╦╩2╛▌═°í┐_2017.docx]

各位代表：

　　现在，我代表市人民政府向大会作工作报告，请予审议，并请市政协委员和其他列席人员提出意见。

一、本届政府工作回顾

　　2012年6月以来，我们在中共泰州市委的坚强领导下，坚持以党的十八大和十八届三中、四中、五中、六中全会精神为指导，深入贯彻习近平总书记系列重要讲话特别是视察江苏重要讲话精神，主动适应经济发展新常态，自觉践行新发展理念，持续推进全省转型升级综合改革试点，扎实开展三大主题工作，加快建设“四个名城”，经济社会发展取得显著成就。

　　坚持稳中求进，综合实力迈上新台阶。坚定不移把发展作为第一要务，制定出台工业10条、开放10条、人才10条、金融10条等政策措施，在转型升级中保持经济平稳较快发展。经济总量持续攀升。2016年，预计实现地区生产总值4060亿元，五年连跨两个千亿元台阶，人均地区生产总值突破8万元。一般公共预算收入327.6亿元，年均增长8.5%。固定资产投资3170亿元，五年累计突破1万亿元。规模以上工业增加值2750亿元，年均增长12.2%。高新技术产业产值突破5000亿元，占全省比重较2011年提高2.5个百分点。社会消费品零售总额1115亿元，年均增长11.8%。居民消费价格涨幅保持在2%左右。发展层次加快提升。我市被定位为长三角Ⅱ型大城市，全域纳入沿海开发战略。成为长江经济带大健康产业集聚发展试点，创成国家中医药综合改革试验区、康养旅游示范基地。获批国家级金融支持产业转型升级改革创新试验区，建成国家创新型试点城市和知识产权示范城市。获批国家高标准农田建设试点，创成国家现代农业示范区。产业结构进一步优化。三次产业结构由2011年的7.2︰54︰38.8调整为5.9︰48.1︰46。加快发展现代农业，累计新增高标准农田69万亩，综合机械化率80%以上，建成全省首家“亩产吨粮市”，姜堰荣获全国农业改革与建设试点三年总结考核第一名。聚焦聚力三大战略性主导产业，生物医药产销规模全省领先，造船完工量占全国比重达16%，节能与新能源产业规模超过1100亿元。实施传统产业改造提升计划，推进千企升级百企示范工程，累计新增销售10亿元以上企业113家、利税过亿元企业144家，境内外上市和新三板挂牌企业分别达10家、26家。获批创建全国质量强市示范城市，扬子江药业获中国质量奖提名奖。推进“建筑强市”建设，建筑业总产值累计突破1万亿元，8项承建工程获评“鲁班奖”。“十二五”期间，单位地区生产总值能耗累计下降27.2%，主要污染物排放量削减提前完成省定目标任务。服务业发展明显加快，去年实现增加值1870亿元，占比年均提高1.4个百分点。溱湖风景区升级为国家5A级景区，凤城河风景区创成国家级城市中央休闲区，全市接待游客数和旅游总收入分别是2011年的1.8倍、1.9倍。本外币贷款余额达3721亿元，新引进9家银行业金融机构，全市银行机构数达35家。

　　坚持改革创新，经济发展积蓄新动能。实施全面深化改革两年行动计划，推进以科技创新为核心的全面创新，改革创新成为积聚发展优势的鲜明特征。供给侧结构性改革扎实推进。围绕“三去一降一补”，在全省率先推出30条重点举措，形成“1+25”政策体系。提前完成省下达水泥、钢铁等行业淘汰落后产能任务，化解船舶行业产能143万载重吨；市区商品住宅去化周期缩短至9.5个月；贷款不良率降至1.75%，规模以上工业企业资产负债率降至51%；“营改增”累计减税42.3亿元，去年直接降低企业成本55.1亿元；基础设施、民生保障、公共服务等短板进一步补长。重点领域和关键环节改革不断深化。出台并向社会公布“五张清单”，取消和下放行政审批事项405项。在全省首创“互联网+”联合图审模式，医药高新区行政审批实现“园内事园内结”。在全省率先实现企业登记“三证合一”“一照一码”全覆盖，率先启动个体工商户“两证合一”改革，率先开展排污权有偿使用交易试点。深化投融资体制改革，成立市凤城国资控股有限公司，组建城建基金、产业发展基金和信用担保基金，中小企业转贷资金运作良好。科技创新活力迸发。连续多年入选全国科技进步先进市，研发经费支出占地区生产总值比重达2.45%，新获发明专利授权量是2011年的3.5倍，科技进步贡献率达60.5%。成立三大主导产业研究院，建成“泰科易”网上技术交易平台，推进创新创业离岸孵化工作。建立“金三角”人才柔性创业机制，推行创新券、创业券、购房券取得积极成效，累计新增国家“千人计划”专家61名、省“双创团队”9个。

　　坚持扩大开放，项目建设形成新优势。持续推进项目大突破，深入开展“开发园区追赶跨越三年行动”，开放开发水平进一步提高。有效投资持续扩大。累计新开工亿元以上项目1286个，其中10亿元以上项目97个、50亿元以上项目4个，总投资102亿元的中海油气一体化项目竣工投产，国电泰州电厂二期建成投运；完成产业投资8860亿元，工业技改投资超过4000亿元。严格项目准入和绩效考评，工业用地投资强度是2011年的2.5倍，高耗能行业投资增速明显放缓。开放型经济难中求进。精心组织各类招商活动，累计实际使用外资61.3亿美元，落户世界500强企业18家。多措并举稳定外贸增长，累计净增外贸实绩企业402家，去年实现进出口总额102亿美元。加快企业“走出去”步伐，累计完成外经营业额40.3亿美元。优化口岸通关环境，靖江港获批国家一类独立对外开放口岸，海关、国检、海事、边检为开放开发作出积极贡献。载体建设提档升级。中国医药城步入产业化快车道，建成国家新型工业化示范基地，获批全国战略性新兴产业区域集聚发展试点、海峡两岸（泰州）医药产业合作示范区，签约落户国内外医药企业超过600家。泰州出口加工区创成国家级综合保税区，靖江经济开发区升格为国家级开发区，三个南北共建园区呈现良好发展势头。

　　坚持完善功能，城乡建设实现新提升。遵循城市发展规律，统筹城乡规划建设，创成全国文明城市、国家历史文化名城，国家卫生城市实现市（区）全覆盖，“泰州太美、顺风顺水”成为享誉全国的城市名片。中心城市首位度明显提升。姜堰撤市设区，建成区面积扩展到105平方公里。实施城建新提升两年行动计划，周山河新城开发建设全面提速，泰职院新校区、省泰中新校区、凤凰小学交付使用，“双水绕城”、金融服务区、市人民医院新区医院等重点项目建设步伐加快；稻河古街区、三水湾二期建成开街，评书评话博物馆、盐税博物馆建成开馆，老旧小区、城中村、棚户区、后街背巷等老城改造有序实施；东风路、永定路快速化改造即将完成，一批断头路相继接通。国家生态市创建通过考核验收，水生态文明城市建设试点工作全面展开，累计新增造林面积34.6万亩，市区“空转绿”新增绿地面积302万平方米。新型城镇化和城乡一体化稳步推进。实施“城乡转型2115计划”和“美丽城乡建设行动”，城镇化率提高到62.8%，7个镇入选全国重点镇。城市环境综合整治项目全面完成，村庄环境整治实现全覆盖。办好新一轮农村实事，农村饮水安全工程累计新增受益人口120万人，新改建农村道路1780公里、桥梁3340座，疏浚县乡村河道4900条。重大基础设施建设实现突破。泰州大桥通车，扬泰机场通航，宁启铁路复线开通动车，盐泰锡常宜城际铁路、北沿江高铁前期工作取得实质性进展。泰镇高速泰州段建成通车，阜兴泰高速兴化至泰州段、京沪高速江广段扩容工程加快推进。泰州港跻身全国港口20强，核心港区5万吨级集装箱码头开港运营。马甸水利枢纽、川东港整治等重大水利工程加快实施，卤汀河、泰东河、引江河二期等项目顺利竣工。

　　坚持发展为民，社会事业取得新进步。大力实施民生幸福工程，每年将一般公共预算支出的75%、新增财力的80%以上用于改善民生，城乡居民人均可支配收入分别达36750元、17850元，年均分别增长9.6%、10.7%。就业和保障水平不断提升。获评全国创业先进城市，城镇登记失业率保持在3%以下。建立全民参保登记动态管理机制，社会保险主要险种覆盖率稳定在97%以上，城乡低保标准、城镇居民医保和新农合补助标准逐年提高。深入实施精准扶贫，全市人均年收入5000元以下的建档立卡低收入人口实现脱贫。累计新开工各类保障性住房7.4万套，住房保障体系健全率达91.3%。公共服务水平不断提升。办好人民满意教育，终身教育体系发展指数居全省前列，泰州学院成功获批，全域建成国家义务教育发展基本均衡市（区）。医药卫生体制改革深入推进，公立医院全部取消药品加成，县域三级医院实现全覆盖。扎实推进公共文化服务设施建设，创新推出文化惠民券，获评“中国吉祥文化之乡”，成功举办梅兰芳艺术节、建市20周年成就展等系列活动。《泰州市志》出版发行。各市（区）均建成省级公共体育服务体系示范区，成功举办一系列国际国内重大赛事。优化养老服务供给，每千名老年人拥有养老床位37.5张。建成“全国残疾人之家”，在里约残奥会上我市运动员取得历史最好成绩。优先发展公共交通，公共自行车系统建成投用，市区公交出行分担率达25.2%，镇村公交覆盖率达74%。民族宗教、社会科学、广播电视、妇女儿童工作取得新进步，统计、气象、防震、民防、台务、外事侨务及对口支援工作取得新成绩。社会治理水平不断提升。深化法治泰州、平安泰州建设，创成全国“六五”普法先进市，连续五年获评全省综治平安建设先进市，群众安全感保持全省前列。加强领导干部接访下访和社会稳定风险评估，率先实施信访权益保障卡制度，有效预防和化解社会矛盾。应急管理体系不断完善，安全生产形势总体稳定，食品药品安全监管体制进一步理顺。国防后备力量建设和双拥共建工作深入开展，荣获全国双拥模范城“四连冠”。

　　坚持严格履职，自身建设得到新加强。推进依法行政。强化法治政府建设，遵循重大行政决策程序，严格政府规范性文件制定程序，建立政府法律顾问制度。深化政务公开，健全政府信息发布制度，推动行政权力网上公开透明运行。加强行政执法监督，全面落实执法责任制和责任追究制。自觉接受人大的法律监督、工作监督和政协的民主监督，广泛听取各民主党派、工商联、无党派人士和社会各界意见。认真办理人大代表建议和政协提案，落实率超过70%、满意率95%。转变工作作风。弘扬“四个长期”“三个不相信”的新时期泰州精神，践行“马上就办、真抓实干”，坚决把市委决策部署、各项政策措施落到实处。在全省率先推行“互联网+政务服务”，整合开通“12345·政风行风热线”，认真办好市长信箱、望海楼论坛，及时解决基层反映的突出问题。构建“三服务”长效机制，定期组织企业家座谈，打造“包办墙外事、帮办墙内事”服务品牌。加强廉政建设。深入贯彻中央八项规定和省、市委十项规定，扎实开展党的群众路线教育实践活动、“三严三实”专题教育和“两学一做”学习教育，持之以恒纠正“四风”。聚焦重点领域、关键环节和公共资金，加强行政监察和审计监督。落实容错纠错实施办法和“五个激励”“蜗牛奖”制度，建立绩效管理评价体系，整治为官不为和“庸懒散”现象。全面落实党风廉政建设责任制，完善廉政风险防控机制，树立为民务实清廉形象。

　　各位代表，本届政府任职以来取得的成绩，是全市人民在中共泰州市委领导下团结拼搏、奋发作为的结果，与市人大、市政协的监督支持密不可分。在此，我代表市人民政府，向全市广大干部群众，向全体人大代表、政协委员，向各民主党派、工商联、无党派人士、各人民团体和广大离退休老同志，向中央和省驻泰机构以及驻泰部队指战员、武警官兵和政法干警，向所有关心支持泰州发展的海内外朋友们，表示衷心的感谢！

　　我们也清醒地看到，全市经济社会发展仍存在一些问题和不足。船舶、化工等传统产业转型任务艰巨，不少中小微企业面临融资难、盈利难的困境，高水平研发机构、高层次创新人才、高新技术企业总量偏少，出口产品结构、对外贸易方式亟需调整优化，提高转型与创新含金量还要下更大功夫。老城区的道路交通、市政设施、人居环境有待进一步改善，新城区的现代化功能品质有待加快提升，打造宜居幸福城市仍需付出艰辛努力。民生实事与群众需求联系还不够紧密，城乡居民收入与经济发展水平还不匹配，教育医疗养老等公共服务供给还存在不少短板，空气、水、土壤污染治理还需加大力度，提高人民群众幸福感必须贯穿各项工作始终。政府职能转变还不到位，权责脱节、推诿扯皮现象依然存在，依法行政能力仍显不足，加强作风建设永远在路上。

二、今后五年目标任务

　　各位代表，今后五年是泰州发展的转型期、改革的攻坚期、小康的决胜期，也是全市上下奋力打拼“两聚一高”过硬成果的重要战略机遇期。我们要在以习近平同志为核心的党中央坚强领导下，高举中国特色社会主义伟大旗帜，以邓小平理论、“三个代表”重要思想、科学发展观为指导，深入贯彻习近平总书记系列重要讲话精神，紧紧围绕“五位一体”总体布局和“四个全面”战略布局，牢固树立和贯彻落实新发展理念，适应把握引领经济发展新常态，坚定不移地把高水平全面建成小康社会作为核心任务，把“四个名城”建设作为战略支点，把三大主题工作作为主要抓手，把新时期泰州精神作为动力之源，努力建设经济强、百姓富、环境美、社会文明程度高的新泰州。

　　——建设经济强的新泰州。到2021年，经济总量力争“省内进前8、全国40强”，地区生产总值年均增长8%以上，迈上6000亿元新台阶，居民收入、企业利润、财政收入“三个口袋”更加充实。研发经费支出占比2.8%以上，高新技术产业产值占比45%以上，科技进步贡献率62%以上，建成国家创新型城市。形成中国医药城在全国生物医药发展的领先地位，巩固全国最大船舶出口基地的领航地位，确立节能与新能源产业在全省的领军地位，在以产业转型带动经济社会发展转型上取得重大突破。

　　——建设百姓富的新泰州。努力实现居民收入高、就业创业广、社会保障好、教育质量优、医疗保健强的目标，人民群众物质生活殷实、精神生活丰富，全面小康有更广代表性、更多获得感、更高满意度。新增就业30万人、创业25万人，城镇登记失业率控制在3.5%以内；城乡基本社会保险覆盖率超过98%；教育主要发展指标达到现代化水平，义务教育巩固率达100%；“健康泰州”建设深入推进，居民健康主要指标达到国际先进水平。

　　——建设环境美的新泰州。生态文明建设取得重大进展，单位地区生产总值能耗、二氧化碳和主要污染物排放总量明显下降，林木覆盖率达24%，Ⅲ类以上地表水比例每年提高2个百分点，空气质量优良天数比例超过75%，争创国家生态文明建设示范区，彰显自然环境之美、景观特色之美、文化交融之美、城乡协调之美。

　　——建设社会文明程度高的新泰州。中国梦和社会主义核心价值观深入人心，公民文明素质和社会文明程度显著提高，实现全国文明城市“三连冠”。公共文化服务体系更加完善，文化事业文化产业繁荣发展，国家历史文化名城的知名度美誉度明显提升。文化惠民券累计发放6000万元，文化产业增加值占比提高到6%。法治泰州、平安泰州建设深入推进，法治政府建设初见成效，公共安全体系更加健全，社会公平正义得到保障，诚信体系建设扎实推进，社会治理现代化水平进一步提升，社会既保持和谐稳定又充满旺盛活力。

　　今后五年的工作原则是：肩负起高水平全面建成小康社会的历史使命，将聚力创新作为提升发展水平的核心战略，将聚焦富民作为实现全面小康的根本要求，致力追求更具转型与创新含金量、让人民群众更有获得感的高点定位、增长速度、争先进位。

　　——提高转型与创新含金量、增强人民群众获得感，必须坚持“高水平”这一奋斗指向。围绕建成贯彻新发展理念、体现“强富美高”要求、惠及全市人民的小康社会，在发展速度、发展目标、发展层次上确立更高参照系，能快则快、能超则超、能好则好，努力实现“三赶超三领先”。围绕用好长江经济带大健康产业集聚发展试点、金融支持产业转型升级改革创新试验区等一系列战略机遇，持续推进全省转型升级综合改革试点，努力创造具有泰州特色的改革成果和发展模式。围绕最应干、能够干、必须干好的事情，扎实推进三大主题工作，加快建设“四个名城”，在提高整体发展水平的同时打造亮点、形成标志。

　　——提高转型与创新含金量、增强人民群众获得感，必须坚定“聚力创新”这一工作导向。以全面深化改革推动经济转型升级，在创新文化和创新环境、创新主体和创新人才、创新平台和创新机制上聚力用力，催生更多的新技术、新产业、新模式。以提高开放水平集聚高端资源要素，大力招引能产生先进生产力的优质项目，确保招商有科技含量、转型有优质增量、创新有产业力量。以加快创新驱动推进跨界深度融合，抓住科技创新这个“牛鼻子”，培育创新集群，发展高端产业，形成以创新为引领的经济体系和发展方式。

　　——提高转型与创新含金量、增强人民群众获得感，必须坚守“聚焦富民”这一发展取向。聚焦居民增收难的短板，突出技能型人才、大学生、新型职业农民、增收困难群体等重点人群，拓宽创业、就业、投资等增收渠道，让老百姓的腰包鼓起来。聚焦生态建设欠账多的短板，着力加强生态环境保护和治理，增加生态产品有效供给，让老百姓的生活环境美起来。聚焦教育医疗养老等方面的短板，集中力量办好一批群众看得见、摸得着的民生实事，解决一批事关群众切身利益的民生问题，让老百姓的获得感和幸福感强起来。

　　今后五年的主要任务是：

　　1. 着力推动经济转型升级。牢牢把握供给侧结构性改革这条主线，以打造“品质泰州”为追求，构建以大健康产业为特色、三大主导产业为引领、先进制造业和现代服务业为主体的现代产业体系。加快产业结构调整，落实《中国制造2025泰州实施纲要》，实施企业互联网化提升计划和制造装备升级计划，到2021年三大主导产业规模超过6000亿元；构建“一核、一带、一组团、一全域”的现代服务业发展新格局，服务业增加值占比达50%以上；新增高标准农田200万亩，确保国家现代农业示范区建设走在全国前列。加快发展动力转换，推进三大主导产业研究院建设，发挥科技广场和“泰科易”平台作用，完善创新创业离岸孵化机制，推进产学研协同创新，全市高新技术企业超过1000家。加快新经济发展，培育壮大数字经济、创意经济、分享经济、新金融，大力实施“互联网+”行动计划，助推产业转型发展；加强质量品牌建设，实施标准引领战略，创成全国质量强市示范城市；放大“三张券”效应，打造人才高地，五年引进高层次创新创业人才1000名。

　　2. 持续发力三大主题工作。以解放思想为先导，打好全面深化改革攻坚战。围绕提高资源配置效率、增强核心竞争力和经济免疫力，认真落实“三去一降一补”重点任务；围绕经济体制改革这个主轴，不断深化行政管理体制、投融资体制、科技体制、国资国企等重点改革。以扩大有效投资为关键，增添经济发展新动能。精准做好激发民资、引进外资、对接央企等各项工作，力争五年实际使用外资100亿美元；持续跟踪项目的投入质量、产出效益，力争累计完成产业投资2万亿元；更加注重严把环保门槛，坚决不在新一轮项目建设中留下任何后遗症。以提升发展品质为重点，打造更具魅力的宜居家园。立足长三角Ⅱ型大城市定位，开展第四轮城市总体规划修编，实施新一轮城建新提升两年行动计划，统筹推进老城新城建设；构建“一大三中九小”新型城镇体系，促进中心城市和中小城市、重点镇、新农村协调发展；全力打胜重大交通基础设施建设大决战，加快迈入“高铁时代”。

　　3. 全面推进“四个名城”建设。找准在全省发展布局中的新定位，立足资源禀赋和发展实际，致力构筑区域竞争新优势。医药名城，以中国医药城为核心支撑，突出“医药养游”融合发展，加强国际医药博览会、泰州医药峰会等重大平台建设，推进医药产业前伸后延、向高端攀升，打造国家级前沿医疗技术应用基地、长三角医养结合养生养老胜地。港口名城，把握“共抓大保护、不搞大开发”的基调，推进长江岸线修复和资源整合，构建衔接畅通的港口集疏运网络，建成综合保税区B区，形成港产城联动发展格局。文化名城，聚焦“三强两高”目标，提升文化软实力，打造水文化、大健康文化、吉祥文化等特色品牌，彰显“泰州太美、顺风顺水”城市内涵魅力。生态名城，坚持“绿水青山就是金山银山”，以“两减六治三提升”专项行动为抓手，落实主体功能区规划，加大“绿色调控”力度，完善环境保护长效机制，力争实现生态环境质量的根本性好转。

　　4. 共建共享幸福美好家园。自觉践行以人民为中心的发展思想，深入实施“七大公共服务体系提升工程”，让人民群众过上好日子。坚持富民为先，开展“创业泰州”八大行动，推进农村产权制度改革，实施脱贫致富奔小康工程，进一步增加工资性收入、提高经营性收入、扩大财产性收入、巩固转移性收入。坚持均衡为要，加快构建“公平普惠、优质多样、充满活力、人民满意”的卓越教育体系，“健身、防病、治病一体化”的全民健康体系，更加公平、更可持续的社会保障体系。坚持和谐为基，强化系统治理、依法治理、综合治理、源头治理，织密现代社会治安防控网、矛盾纠纷预防化解网、基础综合服务管理网，打造全省平安建设示范区、法治建设先导区、社会治理样板区。

　　各位代表，建设“强富美高”新泰州、高水平全面建成小康社会是我们必须肩负的重大责任。与500万怀揣激情梦想的泰州人民加油干，同500万拥有智慧力量的泰州人民齐打拼，我们一定能交出无愧于时代的合格答卷，开创更加美好的明天！

三、2017年工作建议

　　五年看头年，实绩靠实干。今年是党的十九大召开之年，是供给侧结构性改革的深化之年，也是新一届政府的开局之年。做好今年工作，具有特别重要的意义。

　　今年政府工作的总体要求是：全面贯彻党的十八大和十八届三中、四中、五中、六中全会精神，认真落实省第十三次党代会、市第五次党代会和市委五届二次全会决策部署，坚持稳中求进工作总基调，以提高发展质量和效益为中心，以推进供给侧结构性改革为主线，突出“两聚一高”发展布局，深入推进三大主题工作，全面做好稳增长、促改革、调结构、惠民生、优生态、防风险各项工作，在建设“强富美高”新泰州征程上迈出坚实步伐，以优异成绩迎接党的十九大胜利召开。

　　今年全市国民经济和社会发展主要预期目标建议安排为：地区生产总值增长8%以上，一般公共预算收入保持正增长，固定资产投资增长12%左右，社会消费品零售总额增长11%左右，城乡居民收入实现与经济基本同步增长，居民消费价格涨幅3%左右，城镇登记失业率控制在3%以内，研发经费支出占比2.5%左右，节能减排完成省定目标任务。

　　今年，建议重点抓好十个方面的工作：

　　1. 构筑现代产业高地。围绕提升三大主导产业国际竞争力，推进集聚发展、转型发展、创新发展，力争产业规模达3500亿元。坚持以中国医药城为龙头，加强生物医药创新研究院、创新中心及免疫技术服务、安评中心等载体平台建设，全力推进大健康产业集聚发展试点，加快中国医药城整体上市步伐。引导船舶行业抢抓军民融合机遇，加快开发具有自主知识产权的“两高”船型，有效化解接单难、交船难。支持新能源重点企业领衔制定行业标准，推动组建动力电池研究院等研发载体，做强国家级石墨烯研究检测平台。突出“四换”并进改造提升传统产业，实施“两化融合”示范行动和“机器换人”三年行动计划，力争全市工业生产装备和智能制造水平进入全省第一方阵。推进建筑产业现代化，加快建设“建筑强市”。统筹发展生产性服务业和生活性服务业，抓好现代商贸、科技金融、现代物流等重点产业发展，培育电子商务、云制造研发服务等产业，做大做强“网上中国医药城”、综保区跨境电商等平台。启动全域旅游建设，支持凤城河风景区创建国家5A级景区、溱湖风景区创建国家级旅游度假区。积极推进农业供给侧结构性改革，加快转变农业发展方式。大力调整农业结构，促进一二三产融合发展。推广“姜堰桥头经验”，结合农村土地“三权分置”改革，发展以家庭农场为主的多种形式的适度规模经营。加快姜堰、兴化高标准农田试点项目建设，新增高标准农田25万亩。加强农业全产业链标准化、品牌化建设，种植业“三品”认证比重提高到50%。实施农业技术推广计划，推进粮食生产全程机械化，推广应用智慧农业服务平台，支持市农业开发区创建国家农业物联网工程技术中心。深化供销合作社综合改革，不断提升为农服务能力。

　　2. 支持实体经济振兴。合理引导发展预期。推进工业企业大数据平台建设，强化对重点行业、企业、指标的监测分析，抓好30个重大增长点的跟踪服务，确保规模以上工业企业达3000家、利润增长10%。引导企业发扬“工匠精神”，加强全面质量管理和品牌建设，扩大高质量产品和服务供给。集成整合扶持企业发展的各类政策，落实好减税降费各项举措，让企业感受更直接、政策利好更普惠。推进社会信用体系建设，搭建全社会信用信息服务平台，健全守信激励、失信惩戒机制。强化要素科学保障。实施差别化信贷政策，完善中小企业转贷资金市区联动机制，创新小微企业融资产品服务模式，建成产融综合服务中心，让更多金融活水流入实体经济。立足土地节约集约利用“双提升”，推广“321”综合节地模式，细化产业用地分类考核标准，探索租让并举、弹性出让等工业用地供应方式创新。推进“才富泰州”平台建设，加强高层次创新人才引进培养，实施“蓝领精英培育工程”，促进人力资源合理流动配置。营造良好营商环境。落实“亲”“清”要求，坚持企业家座谈会制度，深化“三服务”活动，当好服务发展的“店小二”。实施企业家“三百”培育工程，组织“功勋企业家”评选表彰，保护和激发企业家精神。支持企业家专心创新创业，切实维护公平、保护产权、呵护市场秩序。

　　3. 加大有效投资力度。注重实施精准招商。以三大主导产业为重点，瞄准世界500强、国内500强和行业100强企业，加强龙头型、配套型项目招引。精心组织“2266”招商计划，强化园区招商考核，组建市直属招商局，开展产业链招商、互联网招商、驻点招商和以商引商，力争新签约亿元以上项目超过800个。加快项目落地见效。建立项目建设分级督查推进和协调服务机制，完善重大工业项目组织推进体系，力争产业投资增长18%，新开工亿元以上项目超过500个、竣工400个。加大对本土企业新上项目、增资扩股、并购重组的支持力度，组织实施一批重点技改项目，力争完成工业技改投资1300亿元。按照“四个决不允许”的要求严格环保准入，对亿元以上新开工项目实行全程跟踪考核，确保项目建设优质高效。提升园区发展水平。支持现有省级开发园区整合重构、错位竞争，探索飞地开发、园区共建、项目转移的有效形式。启动实施高新区培育行动，提升发展2家现有高新区，积极争创4家省级高新区，遴选挂牌4家市级高新区。支持泰兴经济开发区、泰州港经济开发区创建国家级开发区，支持兴化经济开发区创建国家级健康食品产业园区。

　　4. 抓好深化改革工作。深入推进“三去一降一补”。加快“僵尸企业”市场化退出步伐，坚决取缔非法产能，确保完成压减过剩产能和淘汰落后产能目标任务。把去库存和促进人口城镇化、房地产健康稳定发展结合起来，科学供应开发用地，重点解决“四个不平衡”。推动企业深化股改和规范化提升，启动实施百企上市（挂牌）行动计划，支持企业市场化、法治化债转股，非金融企业直接融资占比提高到28%。继续开展降低实体经济企业成本行动，全年直接降低企业成本40亿元。聚焦基础设施、民生保障、生态环境等薄弱环节，保持有效投资强度，增强发展的整体性和协调性。加快推进基础性关键性改革。推进行政管理体制改革，严格落实权力清单和责任清单，开展相对集中行政许可权改革试点，市行政审批局挂牌运行；加快政务服务“一张网”建设，努力做到行政审批网上办、集中批、联合审、区域评、代办制、不见面；全面推行“双随机、一公开”，支持市（区）开展跨部门综合执法。推进金融支持产业转型升级改革创新试验区建设，建成运营“1+10”产业投资基金和国家一类新药投资基金，筹建大健康基金类股权投融撮合平台、金融资产交易平台、金融控股集团。推进国资国企改革，逐步实现国有资产资源统筹管理、规范运作，努力提高国有资本运营效率和管理水平。推进财税体制改革，加强政府性债务管理，完善全口径预算管理体系和财政管理体制。

　　5. 着力强化创新驱动。突出企业主体地位。深入实施高新技术企业千企行动计划和“小升高”专项行动，力争新增高新技术企业200家。引导企业加大研发投入，开展企业研发机构“百企示范、千企试点、万企行动”，新增省级以上研发机构25家。扎实抓好离岸孵化。设立总规模20亿元的离岸孵化基金，加快建设100个离岸孵化基地，新增在孵项目500个左右，通过离岸孵化落户创新型项目300个以上。提高协同创新水平。依托特色产业和技术优势，实施产学研协同创新行动计划，加快国家疫苗工程技术研究中心申报。拓展“泰科易”网上技术交易平台功能，加强与省技术产权交易所、跨国技术转移中心等机构的对接合作。强化产业技术创新战略联盟建设，推进共性关键技术联合攻关，加快形成一批创新成果。完善创新生态体系。对接落实省科技创新“40条”，扩大科技金融风险补偿资金池规模，改进科研项目和资金管理，提高专利培育和知识产权保护水平。发挥三大主导产业研究院作用，加强科技广场、新型创新空间和科技企业孵化器建设，促进创新政策、资源、服务向企业集聚。用好国家级精细化学品、不锈钢制品质检中心，加快国家特种劳动防护产品质检中心创建，打造长江经济带检验检测新高地。推进特色小镇建设。按照“531”总体要求，合理布局功能板块，创新发展特色产业，搭建完善平台载体，确保10个特色小镇建设取得阶段性成果。

　　6. 进一步扩大对外开放。提高利用外资规模和质量。组织开展境外投资促进活动，强化产业链、价值链中高端招商，力争全年实际使用外资16亿美元。推进服务业和制造业扩大开放，发展总部经济和功能性机构，拓宽利用外资领域。鼓励外资企业通过合资、参股、并购等方式与本土企业开展战略合作，实现技术、管理和资本的高位嫁接。推动外贸向优质优价、优进优出转变。加强国家生物制药、新能源科技兴贸创新基地和船舶出口基地建设，支持先进技术设备、关键零部件、能源原材料进口，力争进出口总额实现正增长。优化进出口产品、市场和地区结构，推进加工贸易创新，提升加工贸易增值率和新兴市场、一般贸易、服务贸易出口比重。大力发展新型贸易方式，开展第二批跨境电商产业园和海外仓试点，加快国际服务外包示范区建设、国家药品进口口岸城市创建，积极申报综合保税区B区。主动融入“一带一路”国家战略。制定企业“走出去”产业目录，推动优势产业企业在“一带一路”沿线布局，开展产能和装备制造合作，实施跨国并购和境外上市，增强国际化经营能力。借鉴复制自贸区改革经验，健全同国际投资贸易规则相适应的体制机制，提高投资贸易便利化水平。

　　7. 提升城乡发展品质。聚力解决“欠账短板”。围绕解决交通拥堵的问题，启动S231高架北延、东风路北延、永定路西延、长江大道、站前路快速化改造，首先形成“一环三射一贯通”快速路网；推进京泰路南延、济川路东延、吴陵路北延等主干道路建设，实施道路接通和“微循环”畅通工程，基本消除海陵区主次干道断头路；加强公共停车场地规划建设，市区新增停车泊位5000个；加快智慧交通建设，开展“治堵治乱保畅”专项行动，完善公共自行车、城市道路慢行系统。围绕解决绿化总量不足、分布不均的问题，实施城市绿化“1213”工程，推进重点地块、重要路段绿化提档升级，建成区绿化覆盖率达42%。围绕解决棚户区环境脏乱差的问题，加大分类成片改造和征收建设力度，确保完成棚户区改造三年行动计划。推进老城有机更新。实施“五大城市修补行动”，改造提升西客站、城中、东部片区，加强扬州路、南通路、江洲路等道路沿线出新整治。继续开展老旧小区“微整治”，升级改造低洼易涝地段排水系统。统筹推进水气电、雨污分流、网络通信等管网建设，新建污水管网60公里。加快新城建设步伐。基本形成“路网畅通、出行便利”的新区道路框架，推进金融服务区、医药高新区商务服务中心、华润万象城建设，启动周山河初中、小学及体育公园建设，确保市人民医院新区医院、中心血站和120急救中心建成投用。持续优化城市环境。扎实抓好“双水绕城”及凤凰河、南官河景观提升工程，加快实施天禄湖、凤栖湖建设。加强建筑、餐厨、医疗等垃圾处理设施建设，完善农贸市场、公共厕所、垃圾中转站等市政基础设施。开工建设京泰路地下综合管廊。深化城管执法体制改革，合理划分城市管理事权，提高数字化城市管理水平。推进新型城镇化和城乡发展一体化。抓好国家新型城镇化综合试点，推动农业转移人口市民化，逐步实现城乡基本公共服务均等化。开展“强富美高”新农村示范创建，建成省级美丽乡村14个。办好新一轮农村实事，完成农村公路提档升级280公里、新改建桥梁180座，改造农村危房1500户，实现管道燃气达镇全覆盖。加强重大交通基础设施建设。打好“两高铁、两通道”攻坚战，全力争取盐泰锡常宜城际铁路开工建设，同步推进江阴靖江第二过江通道建设，北沿江高铁、泰常过江通道前期工作取得重大突破。建成兴泰高速、京沪高速江广扩容段，开展阜兴泰高速二期、宁通高速广陵至南通段扩容、高兴东高速前期工作。组建港口建设发展有限公司，推进核心港区集装箱码头和集疏运体系建设，启动建设港口物流园区。

　　8. 推动文化繁荣发展。深化省级公共文化服务体系示范市创建，加快建设城市“15分钟文化圈”、农村“十里文化圈”，提高图书馆、博物馆、美术馆资源数字化水平。发挥文化惠民券引导作用，建立政府购买公共文化服务机制，持续开展“百团千场”“百姓大舞台”等群众性文化活动。加快“书香泰州”建设，普及科学文化知识。按照“1133”文化产业布局，依托互联网、金融、科技、旅游等载体资源发展文化新业态，培育吉祥文化产业集群，发展创意文化产业。加强历史文化资源保护、传承和利用，推进涵东等历史文化街区有序保护更新，开展宫氏住宅、笔颖楼、万字会等文保建筑修缮。实施文化精品创作工程和优秀传统文化传承计划，加强文化人才队伍建设，努力多出脍炙人口、深入人心的精品力作。完善文明城市创建长效机制，深入开展社会主义核心价值观“五进”、未成年人道德实践和美德善行系列活动，不断提升社会文明程度。

　　9. 加强生态文明建设。启动生态文明建设示范区创建，实施“两减六治三提升”专项行动，集中力量解决环境突出问题；深化生态文明体制改革，严格落实生态红线保护制度，放大绿色考核差别化试点效应，完善绿色金融政策体系。更大力度治理空气雾霾，采取控煤、减排、降尘、秸秆禁烧等综合治理措施，确保二级以上空气质量天数比例达70%。推进国家水生态文明城市建设试点，全面推行河长制，继续开展城市黑臭河道治理，确保地表水省控断面优于Ⅲ类水质比例达83%以上。加强土壤污染治理与修复，系统整治农业面源、重金属和工业固废污染。全力保障区域生态环境安全，加快打造中心城市北部和沿江地区生态走廊，着力构建覆盖城乡的绿色网络和绿化体系；加强沿江环境污染综合治理，设立沿江化工产业转型基金和专项保险，强力推进化工企业两年集中整治行动。开展第二次全国污染源普查，强化环保联动执法，最大限度消除环境风险隐患。

　　10. 切实增进民生福祉。力促居民收入持续较快增长。出台“创业富民30条”，鼓励支持科技人员、大学生、城镇居民、返乡农民自主创业，确保新增创业5万人。加快建设众创空间、创业孵化基地、农村创业就业服务中心，引导和支持小微创业者在“双创”中实现创收致富，努力扩大中等收入群体。把就业作为民生之本，坚持产业升级和扩大就业规模、调整就业结构联动推进，积极拓展就业新空间。加强就业困难人员动态管理和去产能转岗职工的免费培训，新增城镇就业6万人以上。健全职工工资正常增长机制，着力构建和谐劳动关系。提高企业职工养老保险统筹层次，推进机关事业单位养老保险制度改革，开展生育保险和基本医疗保险合并试点。完善最低生活保障制度，加快健全“救急难”机制，支持残疾人和慈善福利事业发展。聚焦黄桥老区和里下河经济薄弱地区，抓好新一轮脱贫攻坚，确保50%的建档立卡低收入农户和经济薄弱村实现脱贫。提高公共服务供给水平。推进教育现代化，深化教育改革，推广教育信息化应用模式，深入实施素质教育“5+2”工程，加快缩小城乡、区域、校际教育差距。健全现代医疗卫生体系，完善基本医疗卫生制度，加快市妇幼保健院、精神卫生中心建设，确保市中医院新院建成投用。扶持发展养老事业，推进农村区域性养老中心、政府兜底型养老机构、标准化社区养老服务中心和日间照料中心建设。贯彻国家生育政策，推进服务资源整合，提高妇女儿童健康水平和出生人口素质。构建全民健身服务网络，倡导健康生活方式。完成首批市民卡发行，逐步扩大使用范围。加强和创新社会治理。树立治安环境也是投资竞争力的理念，争创全国社会治安综合治理先进市。健全立体化、信息化社会治安防控体系，加强对多发性侵财犯罪、涉网新型犯罪的打击治理。开展信访积案“双百攻坚”，落实重大决策社会稳定风险评估机制。深入实施“七五”普法，加强法律援助工作。严格落实安全生产责任制，突出抓好危险化学品、交通运输、建筑施工等重点行业领域专项整治，坚决防范重特大事故发生。推进应急指挥平台和管理体系建设，加强食品药品安全监管和综合治理，保障人民群众生命安全和身体健康。

四、加强政府自身建设

　　新形势新任务对政府自身建设提出了新的更高要求，必须紧扣“三个不变”“四个转变”的总体思路和政府治理能力现代化的目标，更加务实、更高效率、更有担当地做好政府工作。

　　弘扬求真务实作风。践行新发展理念，善用改革的思维，相信市场的力量，正确处理好速度与质量、当前与长远的关系，致力追求“金色GDP、绿色GDP、幸福GDP”。牢记为民宗旨，扎实开展“百团千村万企大走访大落实”活动，在接地气、抓具体中推动政策举措落地生根。深入调查研究，加强工作的预见性、计划性、统筹性，保持定力和韧劲，不浮躁，不折腾。坚持问题导向，不绕圈子直面挑战，撸起袖子攻坚克难，切实把发展最急迫的事办好、把企业最反感的事改好、把群众最期盼的事做好。

　　转变政府工作方式。围绕科学民主决策，严格落实公众参与、专家论证、风险评估、合法性审查、集体讨论决定等法定程序，确保决策制度科学、程序正当、过程公开、责任明确。围绕权力规范运行，落实法治政府建设纲要，将行政权力纳入法律框架下、制度笼子里和平台监控中，进一步增加透明度、提高公信力。围绕提升工作效能，发扬钉钉子精神，对各项重点工作实行项目化推进，抓好常态化督查。围绕方便群众办事，建成“互联网+政务服务”体系，促进实体大厅与网上平台互动融合，让居民和企业少跑腿、好办事。

　　加强勤政廉政建设。保持“激情燃烧、干事创业”的精神状态，整肃“能为不为、敷衍了事”的怠政行为，力促勤勉敬业、守土尽责。健全激励和容错纠错机制，为改革创新者撑腰鼓劲，营造想干事、能干事、干成事的浓厚氛围。深入贯彻中央八项规定和省、市委十项规定，持续纠正“四风”，严控“三公”经费，降低行政成本。自觉接受人大法律监督、工作监督和政协民主监督，更加重视审计监督和舆论监督，及时回应社会关切。认真落实党风廉政建设责任制，完善政府系统惩治和预防腐败体系。加强政府公务人员培训，巩固“两学一做”学习教育成果，提高政治素质和业务能力，打造一支忠诚干净担当的泰州铁军。

　　各位代表，高水平全面建成小康社会的光荣使命，引领我们砥砺奋进；全市人民的信任与期盼，鞭策我们勇往直前。让我们更加紧密地团结在以习近平同志为核心的党中央周围，在省委省政府和市委的坚强领导下，紧紧依靠全市广大干部群众，解放思想，开拓创新，夙夜在公，真抓实干，为建设“强富美高”新泰州而不懈奋斗！
